# Did social isolation affect anxiety and sleep quality of elite soccer players during the COVID-19 lockdown? Comparisons to training before distancing in the pandemic and outlook for mental health

**DOI:** 10.3389/fpsyg.2024.1490862

**Published:** 2024-11-29

**Authors:** Whyllerton Mayron da Cruz, Danilo Reis Coimbra, Guilherme Torres Vilarino, Amândio Manuel Cupido dos Santos, Vernon Furtado da Silva, Stefania Mancone, Lavinia Falese, Pierluigi Diotaiuti, Alexandro Andrade

**Affiliations:** ^1^Center of Health and Sport Science—CEFID, Santa Catarina State University-UDESC, Florianópolis, Brazil; ^2^Department of Physical Education, Federal University of Juiz de Fora, Juiz de Fora, Brazil; ^3^Faculty of Sport Sciences and Physical Education, University of Coimbra, Coimbra, Portugal; ^4^Federal University of Rio de Janeiro (UFRJ), Rio de Janeiro, Brazil and Visiting Professor, Department of Physical Education, Federal University of Rondônia, (UNIR), Porto Velho, Brazil; ^5^Department of Human Sciences, Society and Health, University of Cassino and Southern Lazio, Cassino, Italy

**Keywords:** soccer, high performance, detraining, psychological aspects, mental health, COVID-19

## Abstract

**Objective:**

To analyze the anxiety levels and sleep quality of elite soccer athletes in training pre-pandemic and during the lockdown caused by COVID-19.

**Method:**

This is an exploratory study with a longitudinal design carried out with elite soccer athletes from two Brazilian soccer clubs. Data collection took place in person pre-pandemic (training) and online (during lockdown) between February and May 2020. The instruments used to assess sleep, daytime sleepiness, and anxiety were the Pittsburgh Sleep Quality Index (PSQI), Epworth Daytime Sleepiness Scale-(ESS-BR), and Competitive Anxiety Scale (SCAT). For data analysis, descriptive statistics (frequencies, percentages, maximum and minimum) and non-parametric inferential statistics were used, establishing a significance of *p* < 0.05.

**Results:**

In total, 76 male soccer athletes participated in the study. A significant increase was observed in anxiety levels in confinement compared to pre-pandemic training (*p* = 0.017; *g* = 0.83), and sleepiness significantly reduced in training compared to baseline levels (*p* = 0.007; *g* = 0.48). The athletes demonstrated good sleep quality and the pandemic did not significantly alter daytime sleepiness compared to training and baseline.

**Conclusion:**

From the results it can be concluded that elite soccer athletes presented alterations in anxiety levels compared to training during confinement, however, no effects of confinement were observed on sleep quality and sleepiness. New studies are needed to analyze the long-term consequences of the pandemic and the relationships between anxiety and sleep in training and competition in athletes.

## Introduction

High-performance soccer requires a high and intense level of physical, technical, and psychological preparation, demanding intense variability of stimuli and complex preparation (Barnes et al., 2014; Ermidis et al., 2019; Thapa et al., 2021). Interdisciplinary training programs are designed to attend the full preparation of the athlete, aiming at improving or maintaining performance and reducing the risks of injuries and absences for clinical reasons (Iaia et al., 2009; Bowen et al., 2017; Clemente et al., 2021b, 2023; Sarmento et al., 2021).

Thus, during training under normal conditions, the soccer athlete is subjected to several changes in training loads (Jaspers et al., 2017; Impellizzeri et al., 2019), which can cause physical and psychological damage if they exceed the capacity for recovery, a topic widely discussed in high-performance soccer (Orviz-Martínez et al., 2021). In addition to the strong relationship of the impact of training and competitions on the involvement of injuries, which has been widely discussed there is also a role of sleep quality as an attenuating factor in these impairments (Lastella et al., 2016; Poitras et al., 2016; Gledhill et al., 2018; Altarriba-Bartes et al., 2021).

In sports in general, monitoring the effects of training has been associated with promising outcomes, such as the prevention of overtraining, control of stress and anxiety levels, and improvement in overall well-being (Brink et al., 2010; Meeusen et al., 2010; Laux et al., 2015; Dominski et al., 2021; Chang et al., 2020; Jia et al., 2022; Russell et al., 2019). Furthermore, sleep quality is a variable directly related to recovery and sports performance (Polito et al., 2017; Altarriba-Bartes et al., 2021). In a review study, the use of psychometric scales was shown to be fundamental for assessing a variety of psychological and behavioral phenomena. These subjective measures provide a quantitative framework for evaluating traits, attitudes, emotions, and other aspects of athletes (Laux et al., 2015; Saw et al., 2016; Polito et al., 2017). However, it is known that each modality presents specific demands and characteristics, whether from a technical, tactical, or psychological point of view (Thompson et al., 2020). Recently, different psychophysiological outcomes have been analyzed associated with training load, leading to reports of cortisol alterations, caused by high levels of anxiety, and testosterone variations, due to changes in mood states (Jaspers et al., 2017; Slimani et al., 2017; Kunrath et al., 2020).

Thus, an important point for developing more effective training strategies, improving performance, and promoting the integral health of athletes involves understanding the interaction between body regulation and emotional functions. Body and emotional regulation directly impact the performance and health of athletes, contributing to facing the physical and psychological challenges specific to sport (Meeusen et al., 2010; Madsen et al., 2021; Pellino et al., 2022). Studies have shown that the ability to control emotions and maintain balance under pressure optimizes performance and prevents injuries (Haddad et al., 2013; Saw et al., 2016; Impellizzeri et al., 2019; Altarriba-Bartes et al., 2021; Miguel et al., 2021). Emotional regulation is also linked to resilience, general well-being, and recovery, which are essential for a rigorous sports career, especially in high training and competition demands (Bicalho et al., 2020; Ashford et al., 2021).

In 2020 the COVID-19 pandemic affected the general health of different populations around the world (Diotaiuti et al., 2021, 2023; Leguizamo et al., 2021; D’Oliveira et al., 2022; da Cruz et al., 2022; Andrade et al., 2023), including high-performance sports athletes (Andreato et al., 2020; Pété et al., 2022). This population has several significant recovery needs, including sleep and reduced anxiety (Claudino et al., 2021; Coimbra et al., 2021; Ballesio et al., 2022). In soccer, athletes and sports professionals were concerned with the effects of detraining and deconditioning of players, highlighting necessary attention and caution on the return to activities (Mohr et al., 2020; García-Aliaga et al., 2022). Other studies found that confinement and quarantine produced negative effects, such as significant increases in the body fat percentage and reduced physical performance (Grazioli et al., 2020). A recent systematic review found that although strength and endurance levels were maintained through home training programs, a lack of soccer-specific stimuli during confinement may have an impact on the power and speed performance of soccer players (Friebe et al., 2022).

Research has also indicated that factors beyond physical, technical, and tactical aspects, directly and indirectly, affect psychological variables, with increased anxiety and altered sleep quality in athletes in general (Vindegaard and Benros, 2020; Jia et al., 2022). It is known that sleep is fundamental to the physiological, psychological, and mental states of athletes, and sleep disorders in athletes can affect training and competition directly, or indirectly, generating anxiety and further impairing performance due to increased fatigue (Gupta et al., 2017; Doherty et al., 2021; Walsh et al., 2021).

The COVID-19 pandemic had a significant impact on the sleep quality of athletes. Various studies have highlighted the effects of the pandemic on the sleep patterns and quality of both elite and amateur athletes (Pillay et al., 2020; Martínez-Patiño et al., 2021). Research has shown that the pandemic and associated measures such as lockdowns have led to changes in athletes’ sleep patterns, with some experiencing maintenance of quality and others experiencing worsening sleep, as well as increased severity of insomnia and disruptions to circadian rhythm (Keemss et al., 2022; Romdhani et al., 2022a, 2022b; Walsh et al., 2022; Tan et al., 2023). Additionally, the pandemic has been associated with increased stress, anxiety, and worry, which further contributed to sleep disturbances among athletes (Kocevska et al., 2020; Facer-Childs et al., 2021; Melone et al., 2022).

The reduction in physical activity levels during the pandemic has been linked to lower sleep quality and higher insomnia severity in athletes (Walsh et al., 2022; Romdhani et al., 2023). The impact of the pandemic on sleep quality has been found to vary across individuals, depending on factors such as pre-pandemic sleep quality, training intensity, and compliance with COVID-19 measures (Kocevska et al., 2020; Parsak and Saraç, 2022). Moreover, the pandemic has affected the sleep duration of athletes, with some experiencing changes in their sleep patterns, including waking up earlier and sleeping less (Kurniarobbi et al., 2022; Tan et al., 2023). The COVID-19 pandemic posed significant challenges to the sleep quality of athletes, necessitating further research and interventions to support their well-being during these unprecedented times.

Recent studies showed that implications arising from the COVID-19 pandemic may have altered sleep quality, leading to increased anxiety in athletes (Håkansson et al., 2020; Jurecka et al., 2021). Especially in soccer athletes, a negative impact of the COVID-19 pandemic was verified, considering psychological aspects and mental health (Andrade et al., 2024). In this sense, sleep quality assessments before and during the pandemic enable investigation of the impact of training and confinement on the performance of athletes (Andrade et al., 2016, 2018; Keemss et al., 2022; Poitras et al., 2016). Some studies have shown that athletes’ sleep quality worsened during lockdown, while sleep duration increased (Mon-López et al., 2020; Romdhani et al., 2022a, 2023).

The prevalence rates of poor sleep quality and insomnia symptoms among professional soccer players have been reported to be as high as 68.5 and 27%, respectively (Ballesio et al., 2022). The relationship between poor sleep quality and musculoskeletal injuries in soccer players has also been highlighted, emphasizing the importance of addressing sleep quality to prevent injuries (Clemente et al., 2021a).

Although some recent research focused on the implications of the pandemic on the psychological variables and sleep of soccer athletes, many gaps can still be observed, especially considering the specificity of the sport and the characteristics of elite players.

In this sense, the present study aimed to analyze the anxiety levels and sleep quality of elite soccer athletes, comparing the periods before and during the lockdown imposed by the COVID-19 pandemic. Specifically, we sought to verify whether the context of the pandemic had a significant impact on the variables studied, affecting athletes’ sleep and anxiety levels differently. The hypothesis is that social isolation and changes in sports and personal routines due to the pandemic have increased anxiety levels and impaired the sleep quality of elite athletes.

## Materials and methods

This is an exploratory study with a longitudinal design, conducted with athletes from two Brazilian soccer clubs. The study was approved by the Ethics Committee on Research Involving Human Beings by the State University of Santa Catarina, under protocol number 4.898.541/2021 (CAAE: 44696120.1.0000.0118).

### Sample characteristics

#### Population and sample

Soccer players who had undergone intensive training before the period of social isolation imposed by the pandemic and maintained a regular training routine pre-lockdown and were in quarantine during social distancing were recruited. Participation was voluntary, with informed consent, and data collected included information on their anxiety levels, sleep quality, and general mental health before and during isolation. Demographic data collected to maintain eligibility included age, weight, height, time at the club, years of experience as a football player, hours of sleep, training time during the lockdown, and specific data related to the pandemic, such as hours of sleep and daily training time in isolation. This study did not include clinical aspects, such as possible COVID-19 infection among participants, focusing exclusively on the assessment of anxiety levels related to confinement and the interruption of training and competitions. During training, 76 elite male soccer athletes participated in this research, and during the period of confinement 44 athletes participated in the data collection.

**Figure 1:**
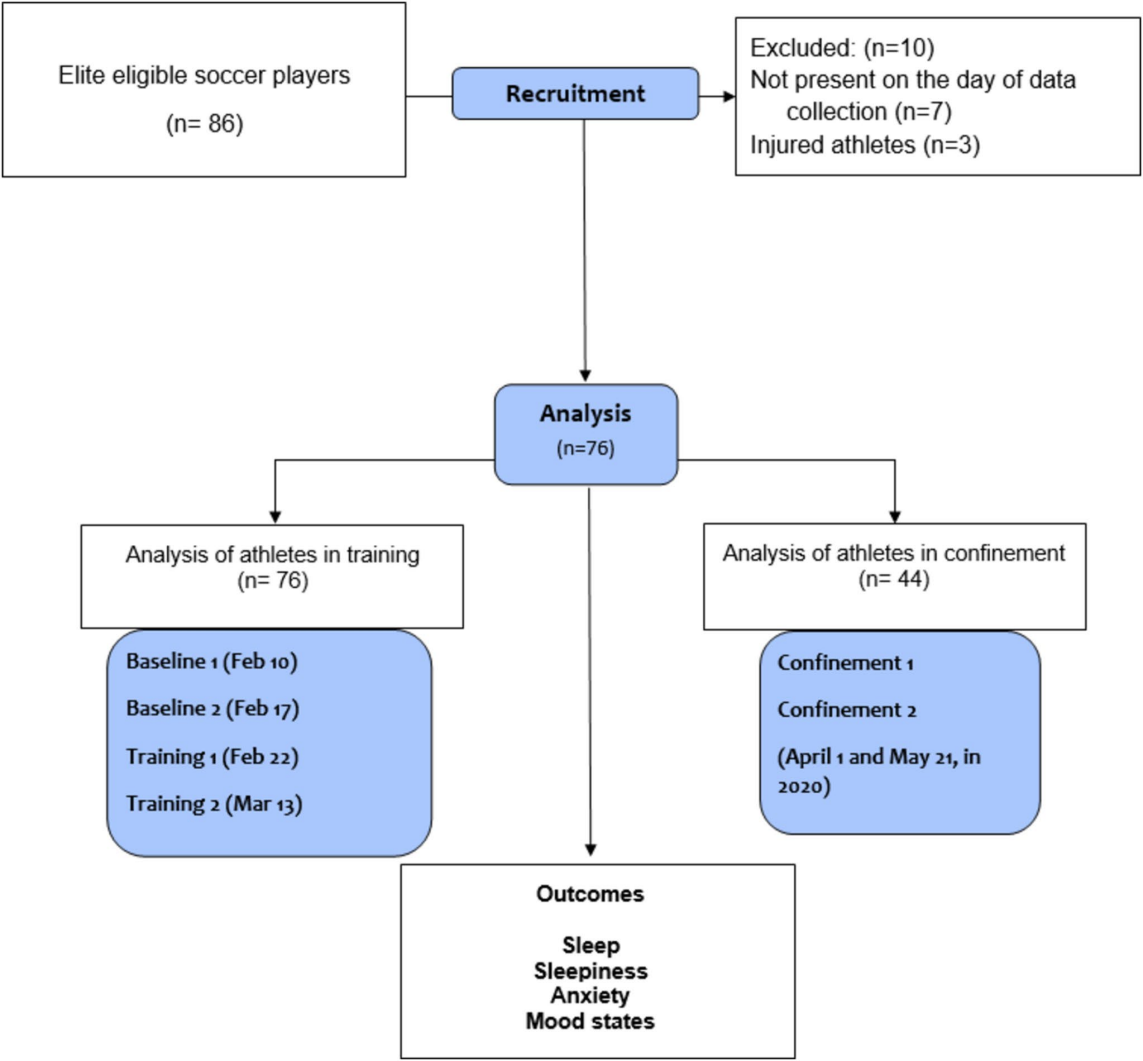
Study flowchart.

This sample loss was justified due to the impossibility of contacting some of the athletes during confinement (Figure 1). All athletes were linked to the Santa Catarina Football Federation—FCF and the Brazilian Football Confederation—CBF.

#### Inclusion criteria

To participate in the study, participants were required to meet the following inclusion criteria:

1. Elite soccer athletes with at least 5 years’ experience in the sport.
2. Not presenting any injury or impediment that would make it impossible to participate in data collection.

#### Exclusion criteria

The following exclusion criteria were followed:

1. Not being a soccer athlete or having minimal experience in the sport.
2. Having a cognitive impairment that hindered answering the research instruments.

### Measures and procedures for data collection

The researchers made institutional contact with the Football Department of two professional soccer teams in the state of Santa Catarina, linked to the Santa Catarina Football Federation (FCF) and the Brazilian Football Confederation (CBF), to present the objectives of the research, followed by the request to carry out data collection with the elite athletes. After an initial meeting, the research was approved by the board of directors and soon after, the date of data collection was scheduled, which took place in person. The athletes’ data were collected after the athletes’ vacation period, during which no training loads were applied. All data collections occurred after signing and delivering the Free and Informed Consent Form (ICF). The athletes received all the necessary information about the research through a face-to-face meeting, previously scheduled, during which they were able to solve all doubts and receive guidance regarding the completion of the research instruments.

During the training, the collections were performed after the end of the weekly training microcycle, denominated training 1 (moderate load) and training 2 (load with higher volume and intensity).

The procedures were carried out in person in the months of February and March 2020 (training) and remotely online in April and May 2020 (athletes in lockdown) due to the COVID-19 pandemic. The researchers remained available during and after the research procedures to clarify any doubts. For all procedures, the guarantees regarding the total confidentiality of identification in the research and information collected were reinforced.

#### Description of activities and training performed by the athletes before lockdown

During the pre-season training period following the holiday period, the players underwent intensive training to improve their physical, technical, and tactical condition. They participated in physical training sessions to increase endurance, strength, and speed, as well as sport-specific skills, such as dribbling, passing, and finishing. Training games were played among the athletes and with the professional team, but no official games were timetabled. These training sessions focused on the application of strategies and tactical training. The players also participated in small-sided game sessions to improve ball possession, switching, and decision-making under pressure. During this period, the coaches assessed the players’ physical and technical conditioning, and experimented with different tactical formations and game strategies, in order to fully prepare for the competitive season.

#### Description of activities and training performed by the athletes during lockdown

During the lockdown caused by the pandemic, the soccer players were subjected to adapted physical training strategies. These included moderate and high intensity training sessions whenever possible, even within the constraints of space and equipment available at home. Flexibility training was emphasized to maintain range of motion and prevent injuries. Isometries were incorporated to strengthen muscles in a static way, using the athlete’s own body weight or household objects as resistance. In addition, functional training was carried out online, guided by videos or instructions from the coaching staff, aiming to replicate specific soccer movements and improve the players’ coordination and agility. The coaching staff prepared detailed training protocols and sent them to the players, ensuring that everyone was aligned with the established objectives and guidelines. These strategies helped players to stay physically fit during lockdown and to prepare for the return to activities.

### Instruments

#### Characterization questionnaire of soccer athletes in pre-pandemic training

This characterization instrument was developed specifically to analyze the characteristics of elite soccer athletes. The instrument is based on questionnaires previously developed with elite athletes of other sports modalities and is composed of open and closed questions (Andrade et al., 2016, 2018).

#### Characterization questionnaire of soccer athletes in lockdown during the COVID-19 pandemic

This instrument was specifically designed to be applied to athletes of different modalities during confinement, and is based on questionnaires that consist of open and closed questions about the athlete’s behavior during confinement.

#### Pittsburgh Sleep Quality Index

The Pittsburgh Sleep Quality Index (PSQI) is an instrument developed with the objective of evaluating the quality of sleep in relation to the previous month, enabling qualitative and quantitative assessment of the quality of sleep. The PSQI was translated and validated for Portuguese and had an overall reliability coefficient (Cronbach’s *α*) of 0.82, indicating a high degree of internal consistency (Bertolazi et al., 2011). The PSQI questions are arranged in seven areas, which can be assigned from zero to three points. The total sum can reach 21 points, and scores higher than 5 points indicate a poor pattern of sleep quality. The results of the nine questions are grouped into seven components: subjective quality, latency, duration, habitual efficiency, disorders, medication use, and daytime dysfunctions. For soccer athletes, the PSQI showed good applicability (Robey et al., 2014; Keemss et al., 2022).

#### Epworth Daytime Sleepiness Scale-(ESE-BR)

The subjective assessment of daytime sleepiness allows the differentiation of individuals with and without excessive sleepiness. The Epworth Scale was used, which consists of eight questions that describe everyday situations that can induce sleepiness with a Cronbach’s alpha of 0.76 (Bertolazi et al., 2009). Each question is graded from zero to three points, with scores above 10 inferring significant daytime sleepiness and above 15 being associated with pathological sleepiness (Lastella et al., 2016; Gwyther et al., 2022).

#### Sport Competition Anxiety Test

The Sport Competition Anxiety Test (SCAT) was used to evaluate the levels of competitive trait anxiety. This scale consists of 15 questions that describe how the individual feels in a given situation. The classification is given in scores, including eight items on activation, two on deactivation, and five on the placebo effect. The score ranges from one to three for the activation items, and from three to one for the deactivation items, and the total score ranges from 10 (low anxiety) to 30 (high anxiety) (de Rose Junior and Vasconcellos, 1997). The internal consistency of the SCAT items was (0.744) (Cronbach’s *α*) above the normal criterion (0.70) (Balamurugan and Saminathan, 2019).

### Data analysis

Data were tabulated and analyzed using the *Statistical Package for Social Science* 20.0^®^ (IBM, United States), licensed from the State University of Santa Catarina—UDESC. Descriptive and inferential statistics were performed. Descriptive statistics were used for exploratory data analysis (frequencies, percentages, means and standard deviation) and the *Kolmogorov*–*Smirnov* test identified that the data do not follow a normal distribution. The outcomes were analyzed using generalized estimation equations (GEE), comparing different moments, baseline 1, baseline 2, 1 week of training, 1 month of training, confinement 1, and confinement 2. The advantage of the GEE method is that when a mediated point is missing, it uses information from an incomplete pair of observations (Zhang et al., 2014). The Bonferroni *post-hoc* test was used to identify the differences between the means in all variables.

## Results

The sample consisted of 76 elite soccer athletes, with a mean age of 18.29 ± 0.96 years. The mean time of experience as a soccer athlete was 6.13 years and the mean hours of sleep was 8.32 h per day before the pandemic and 8.34 h during the pandemic (Table 1).

**Table 1:** Characterization of the sample: anthropometric characteristics, time as an athlete, and permanence in the investigated club (*n* = 76).

Variables	Whole sample (*n* = 76)		
	(Min–Max)		(Mean/SD)
Age (years)	17	20	18.29 (0.96)
Weight (kg)	60	87	74.16 (6.64)
Height (m)	1.70	1.94	1.79 (5.56)
Time at the club (months)	1	84	24.13 (17.01)
Time of experience as a soccer athlete (years)	2	10	6.13 (2.26)
Hours of sleep^a^	6	13	8.32 (1.39)
Pandemic data			
Confinement training time (hours/day) (*n* = 44)	1	3	1.47 (0.55)
Hours of sleep	6	12	8.34 (1.07)

a Hours of sleep before the pandemic.

It was observed that the physical requirement to perform the training routine during the COVID-19 pandemic had little impact, with most of the sample (45.5%) indicating none or low (13.6%) changes in physical demands, however, almost one third of the sample indicated having moderately increased physical demands, and 13.6% of the investigated athletes indicated increases in these efforts (Table 2).

**Table 2:** Perception of physical demand during the confinement of athletes (*n* = 44).

Qualitative variables investigated during the COVID-19 pandemic	None	Low	Moderate	Very much	Extremely
	*n* (%)	*n* (%)	*n* (%)	*n* (%)	*n* (%)
Considering the level of physical demand/effort you put into your routine, how much has this changed in the lockdown period?	20 (45.5)	6 (13.6)	12 (27.3)	2 (4.5)	4 (9.1)

**Figure 2:**
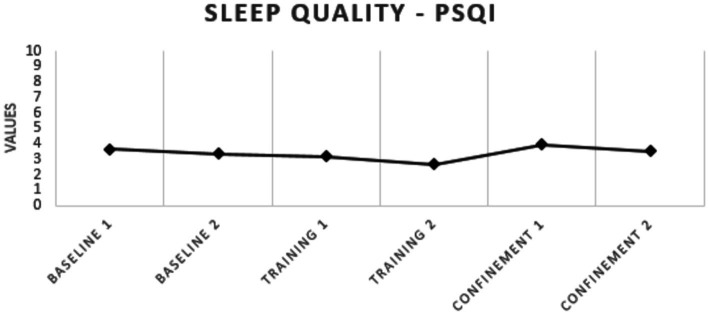
Sleep quality of elite soccer athletes.

**Figure 3:**
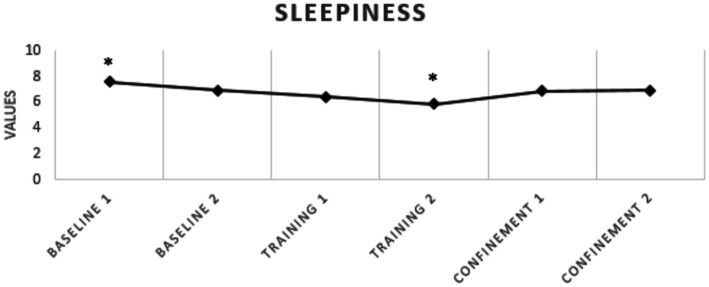
Excessive daytime sleepiness of elite soccer athletes. ^*^Indicates differences between moments *p* ≤ 0.05.

**Figure 4:**
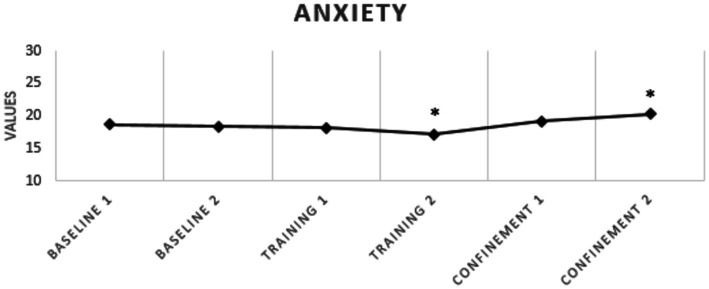
Levels of anxiety of elite soccer athletes. ^*^Indicates differences between moments *p* ≤ 0.05.

Regarding sleep quality, none of the athletes presented poor sleep quality at any of the analyzed moments and there was no significant difference between the analyzed periods (Figure 2). With respect to sleepiness, a significant reduction was observed between baseline 1 and pre-pandemic training 2 (*p* = 0.007; *g* = 0.48), with no increase in sleepiness during confinement (Table 3 and Figure 3). For anxiety, a significant increase was found between training 2 and confinement 2 (*p* = 0.017; *g* = 0.83) (Table 3 and Figure 4).

**Table 3:** Comparison of sleep quality scores, daytime sleepiness, and anxiety at different times during training and confinement.

Variables	Baseline 1	Baseline 2	Training 1	Training 2	Confinement 1	Confinement 2
	Mean ± SD	Mean ± SD	Mean ± SD	Mean ± SD	Mean ± SD	Mean ± SD
	(CI 95%)	(CI 95%)	(CI 95%)	(CI 95%)	(CI 95%)	(CI 95%)
Sleep	3.64 ± 1.70	3.35 ± 1.56	3.17 ± 2.03	2.65 ± 2.00	3.94 ± 2.15	3.50 ± 1.86
	(2.94–4.33)	(2.68–4.02)	(2.25–4.08)	(1.79–3.51)	(2.98–4.91)	(2.68–4.38)
Sleepiness	7.55 ± 3.13^*^	6.94 ± 3.35	6.40 ± 3.79	5.85 ± 3.86^*^	6.86 ± 2.29	6.91 ± 2.81
	(6.75–8.35)	(6.09–7.80)	(5.44–7.35)	(5.74–7.98)	(5.39–7.98)	(5.39–8.43)
Anxiety	18.70 ± 4.02	18.35 ± 3.99	18.15 ± 3.70	17.55 ± 3.52^*^	19.15 ± 3.23	20.26 ± 4.03^*^
	(17.66–19.73)	(17.31–19.39)	(17.20–19.11)	(16.60–18.50)	(17.93–20.37)	(18.74–21.79)

Values obtained using the instruments PSQI, EPWORTH, and SCAT applying generalized estimation equations. ^*^Indicates differences between moments *p* ≤ 0.05.

## Discussion

The current study evaluated the sleep, sleepiness, and anxiety of elite soccer athletes and found that sleep quality was not impaired during the confinement period, despite an increase in anxiety levels. The COVID-19 pandemic impacted the training and competition routine of athletes around the world, changing routines and daily habits (Zinner et al., 2020; Mota et al., 2021). In addition, confinement reduced social and work interactions (Pété et al., 2022). All these changes aroused the interest of the scientific community, which began to investigate the impacts caused by COVID-19 in diverse samples, including soccer athletes (Jia et al., 2022; Andrade et al., 2024).

It is suggested that in a healthy routine, adults sleep for between 6 and 8 h a day, however, professional athletes report sleeping less hours, or present changes in sleep patterns (Meeusen et al., 2010; Lastella et al., 2016; Chang et al., 2020). Due to travel and competitions, many athletes end up sleeping in different locations or have fragmented sleep. The sleep time of the athletes analyzed before the pandemic ranged from 6 to 13 h, indicating that none of the participants sleeps less than recommended (Sargent et al., 2021). In other samples of athletes it was found that the number of hours of sleep positively impacted the recovery of wear and tear caused by sports demands (Di Fronso et al., 2013; Gwyther et al., 2022). During the confinement period, the sleep time was between 6 and 12 h, with no significant differences between the periods. Although some studies show that sleep patterns were modified during the pandemic (Jurecka et al., 2021; Romdhani et al., 2023), our findings corroborate studies in which the assessment during confinement was positive (Keemss et al., 2022). The changes in sleep observed seem to reflect the athletes’ adaptation to this period, with greater flexibility in rest times and the possibility of using personal coping techniques, which may have contributed to better adaptation to confinement (Lin et al., 2017, 2022; Ivarsson et al., 2020; Bernabe-Valero et al., 2021).

In the current study we evaluated sleepiness through the Epworth scale, an instrument used in other studies with athletes (Lastella et al., 2016). The sample of athletes investigated presents good sleep quality, with no negative changes observed in daytime sleepiness, and our data reveal a significant improvement between baseline 1 and training 2. The change between these moments indicates that the practice of physical activity may have contributed to this reduction (Haddad et al., 2013). Excessive daytime sleepiness can be indicative of poor sleep quality, because even when reaching the recommended number of sleep hours, sleep may not be efficient or restorative. The regular practice of physical activity is indicated to reduce sleep disorders, and is even recommended as a form of treatment in some cases (Reilly and Edwards, 2007). The reduction in daytime sleepiness can lead to increased productivity and consequent improvement in performance. In the case of athletes, these factors are fundamental in the training and competitive environment; recently, daytime naps were recommended to improve and reduce the wear of stimuli and training in athletes (Jemal et al., 2022).

A monitored home training program was recently tested and led to significant improvements in physical performance and sleep quality during the COVID-19 lockdown (Keemss et al., 2022). However, it is known that not all athletes were able to remain active during the pandemic, and uncertainties about the future was a factor that showed a negative effect on sleep, with impairments in the perception of exertion (Mon-López et al., 2020). In this sense, it is important to observe sensitive periods and assess to what extent and in which cases physical training should be prioritized over mental and psychological support, consistent with the circumstances and contexts required. There are periods that should be observed with caution, because elite athletes who continued to train in sensitive periods showed a reduction in sleep duration, which can compromise other areas of life besides sport (Trabelsi et al., 2022).

The current study found significant increases in anxiety levels in soccer athletes during lockdown compared to pre-pandemic training (Andrade et al., 2024). Uncertainties about their future, career, and health contributed to increased anxiety levels among athletes (Esteves et al., 2021). The uncertainty surrounding the virus, including its transmission and potential long-term effects, created a sense of unease among soccer players (Jia et al., 2022). The fear of contracting the virus and the subsequent disruption to their training and competition schedules added an additional layer of stress to their already demanding lives as athletes (Wagemans et al., 2021). The restrictions imposed due to the pandemic, such as limited access to training facilities and the absence of spectators during matches, further contributed to the anxiety experienced by soccer athletes (Mohr et al., 2020). The absence of the usual support and encouragement from fans created a sense of emptiness and detachment, affecting the players’ motivation and overall mental well-being (Dönmez et al., 2022). Consequently, the psychological impact of the pandemic on soccer players goes beyond their physical health, with anxiety becoming a significant concern that requires attention and support from various stakeholders in the sport.

Studies have shown that elite athletes, including Olympic and Paralympic athletes, experienced increased anxiety and psychological distress during the pandemic (Clemente-Suárez et al., 2020; Håkansson et al., 2020; Mehrsafar et al., 2021). Professional soccer players also exhibited higher levels of anxiety and depressive symptoms during the COVID-19 emergency period (Gouttebarge et al., 2022). Furthermore, the pandemic led to worse mental and emotional health among athletes, although home training programs and quarantine training camps helped attenuate these effects (Jia et al., 2022). The negative impact of the pandemic on mental health was observed not only in professional athletes but also in young athletes, with a substantial percentage reporting a high impact on their mental health (Pons et al., 2020). Additionally, occupational stress among soccer referees during the early stage of reopening matches led to higher levels of depression, anxiety, and pressure, indicating a negative impact on mental health (Liu et al., 2022).

The perceived social support has been found to play a crucial role in the mental health of college soccer players during the COVID-19 lockdown, with isolation and blockade significantly impacting their mental well-being (Liu et al., 2022). Moreover, the pandemic affected the mental performance and health of Canadian national team athletes, leading to low mood symptoms, anxiety, stress, maladaptive behaviors, and a need for time outside of sport for rest and recovery (Dithurbide et al., 2022). The relationship between mental health and athletic identity has been explored, with strong athletic identity being associated with lower feelings of depression in student athletes (Hagiwara et al., 2021).

Overall, the lockdown period had a significant impact on the mental health of elite athletes, leading to stress, anxiety, and psychological distress (Pellino et al., 2022). The findings suggest that the COVID-19 pandemic posed substantial challenges to the mental well-being of soccer athletes, emphasizing the need for targeted support and interventions to address their unique mental health needs during this unprecedented crisis.

Some studies indicate an association between the levels of anxiety and sleep quality, with one variable directly affecting the other (Melone et al., 2022). In this sense, an alert is raised, because anxiety can directly impact sleep and, consequently, the quality of life and general well-being of athletes (Gouttebarge et al., 2015). The COVID-19 pandemic affected and still directly affects mental health, with higher prevalence of anxiety observed in the general population and in athletes (Arora et al., 2020; Jia et al., 2022).

The way athletes seem to be directly impacted by the pandemic and situations inherent to the sport are individualized and deserve equally personalized responses, especially in the face of the uncertainties caused by mandatory lockdown and isolation. In some studies, the strategies of how athletes faced the challenges resulted in a more positive assessment of mental well-being and less consequent psychological distress (Lima et al., 2021; Merino-Muñoz et al., 2021). The examination of anxiety in soccer athletes can be enriched by contrasting their experiences and perspectives. Through the anxiety levels of different soccer players, researchers can uncover valuable insights into the diverse range of factors that contribute to this psychological state. By carefully analyzing and contrasting these differences, researchers can develop tailored interventions and support systems to help soccer athletes effectively manage their anxiety and enhance their overall performance.

The present study revealed that social isolation during the COVID-19 lockdown increased anxiety and impacted the sleep quality of elite football players. To mitigate these effects, interventions such as regular psychological support and sleep monitoring are essential (Jansen, 2021; Leguizamo et al., 2021; Andrade et al., 2024; Sebri et al., 2024). Stress management strategies, such as mindfulness sessions and mental health professionals’ remote monitoring, help reduce anxiety (Pellino et al., 2022; Listiyandini et al., 2024). Furthermore, adjustments in training routines, including breathing exercises and pre-sleep training techniques, are designed to improve the quality of rest in athletes (Villaseca-Vicuña et al., 2021; Katanic et al., 2022). These strategies proved to be essential for future training programs, highlighting that psychological support and attention to sleep must be incorporated continuously to improve athletes’ performance and mental health (Jordana et al., 2020; Rupprecht et al., 2021).

The team can serve as a significant protective factor against anxiety among soccer athletes during the COVID-19 pandemic. The collective nature of a team provides a sense of belonging and support, which can help alleviate anxiety symptoms. Being part of a team allows athletes to share their concerns, fears, and uncertainties with their teammates, fostering a supportive environment where they can find understanding and empathy. This social support network within the team can act as a buffer against anxiety, as athletes can rely on each other for emotional support and encouragement. Moreover, the team group offers a structured routine and a sense of normalcy amidst the uncertainties brought about by the pandemic.

### Strengths, limitations and future directions

This is a groundbreaking study, which looked at the anxiety levels and sleep quality of elite soccer athletes in training pre-pandemic and during the lockdown caused by COVID-19. The restrictions on access to athletes and the uncertainties caused by the pandemic were factors that hindered and prevented the realization of many empirical studies, which in our research, we were able to overcome. Presenting empirical results obtained within the period of confinement, working with elite soccer clubs and athletes is a differential, adding our results and analyses to the set of scientific literature that intends to better understand the impacts of confinement on athletes. In addition, empirical studies inserted within training and competitions (total ecological validity) are rare and fundamental whenever one seeks to investigate the psychology of elite sport.

However, the lack of detailed information on variables such as training and daily sleep recall, may represent a limitation. Incorporating tools like sleep diaries and sleep monitoring technologies could help minimize this limitation by providing more accurate and reliable data. Furthermore, we must consider that the instrument used to assess anxiety predominantly measures trait anxiety in athletes, a more stable characteristic that is less sensitive to momentary variations, such as changes in routine during confinement. Our choice was made so that prolonged confinement could accentuate this trait anxiety. However, we allow that, to assess the acute and immediate impact of the lack of challenge and training, instruments focused on state anxiety are more appropriate. Therefore, it is essential to carefully consider and control these variables in order to ensure the validity and relevance of the research results.

This study has strengths as it addresses a current topic that is little investigated. We present important results for elite soccer athletes and coaches. However, there are also some limitations, such as sample loss during the confinement period. Most research on the impact of the COVID-19 pandemic on psychological aspects and the mental health of athletes has been carried out using resources remotely with confined athletes. We did not control the environment when the athletes responded to the instruments, however, we provided guidance on personal care and on the right moment for responses. It is recommended that future studies be performed with controlled experimental designs, that prioritize interventions already recognized in sport psychology, aiming at improving the quality of evidence in this area.

## Conclusion

The results indicate that, during the confinement due to COVID-19, elite soccer players presented changes in trait anxiety levels compared to the pre-pandemic period. However, these changes should be interpreted with caution, as they are multifactorial and reflect different aspects of emotional responses to confinement. Furthermore, there were no significant effects of confinement on sleep quality and sleepiness, suggesting a positive adaptation associated with greater flexibility in rest times and the use of personal coping strategies during this period.

## Data Availability

The raw data supporting the conclusions of this article will be made available by the authors, without undue reservation.

## References

[ref1] Altarriba-Bartes A. Peña J. Vicens-Bordas J. Casals M. Peirau X. Calleja-González J. (2021). The use of recovery strategies by Spanish first division soccer teams: a cross-sectional survey. Phys. Sportsmed. 49, 297–307. doi: 10.1080/00913847.2020.1819150, PMID: 32882156

[ref2] Andrade A. Bevilacqua G. G. Coimbra D. R. Pereira F. S. Brandt R. (2016). Sleep quality, mood and performance: a study of elite Brazilian volleyball athletes. J. Sports Sci. Med. 15, 601–605. 27928205 PMC5131213

[ref3] Andrade A. Casagrande P. O. Bevilacqua G. G. Pereira F. S. Alves J. F. Goya A. L.. (2018). Perfil sociodemográfico, socioeconômico e esportivo de tenistas infantojuvenis brasileiros de elite. Movimento 24, 65–78. doi: 10.22456/1982-8918.74041

[ref4] Andrade A. D’Oliveira A. dos Santos K. M. Bastos A. C. R. F. Corrado S. Vilarino G. T.. (2023). Impact of social isolation caused by the COVID-19 pandemic on the mood profile of active and sedentary older adults: physical activity as a protective factor. Front. Public Health 11:1221142. doi: 10.3389/fpubh.2023.1221142, PMID: 37849723 PMC10578610

[ref5] Andrade A. D’Oliveira A. Neiva H. P. Gaertner G. da Cruz W. M. (2024). Impact of the COVID-19 pandemic on the psychological aspects and mental health of elite soccer athletes: a systematic review. Front. Psychol. 14:1295652. doi: 10.3389/fpsyg.2023.1295652, PMID: 38333426 PMC10850388

[ref6] Andreato L. V. Coimbra D. R. Andrade A. (2020). Challenges to athletes during the home confinement caused by the COVID-19 pandemic. Strength Cond. J. 42, 1–5. doi: 10.1519/ssc.0000000000000563

[ref7] Arora T. Grey I. Östlundh L. Lam K. B. H. Omar O. M. Arnone D. (2020). The prevalence of psychological consequences of COVID-19: a systematic review and meta-analysis of observational studies. J. Health Psychol. 27, 805–824. doi: 10.1177/1359105320966639, PMID: 33118376

[ref8] Ashford M. Abraham A. Poolton J. (2021). Understanding a player’s decision-making process in team sports: a systematic review of empirical evidence. Sports 9:65. doi: 10.3390/SPORTS9050065, PMID: 34067590 PMC8156213

[ref9] Balamurugan R. Saminathan V. (2019). Study on sport competition anxiety: a statistical analysis. Int. J. Physiol. Nutr. Phys. Educ. 4, 694–696.

[ref10] Ballesio A. Vacca M. Bacaro V. Benazzi A. De Bartolo P. Alivernini F.. (2022). Psychological correlates of insomnia in professional soccer players: an exploratory study. Eur. J. Sport Sci. 22, 897–905. doi: 10.1080/17461391.2021.1892197, PMID: 33599195

[ref11] Barnes C. Archer D. T. Hogg B. Bush M. Bradley P. S. (2014). The evolution of physical and technical performance parameters in the English Premier League. Int. J. Sports Med. 35, 1095–1100. doi: 10.1055/s-0034-1375695, PMID: 25009969

[ref12] Bernabe-Valero G. Melero-Fuentes D. De Lima Argimon I. I. Gerbino M. (2021). Individual differences facing the COVID-19 pandemic: the role of age, gender, personality, and positive psychology. Front. Psychol. 12:644286. doi: 10.3389/fpsyg.2021.644286, PMID: 33815230 PMC8012731

[ref13] Bertolazi A. N. Fagondes S. C. Hoff L. S. Dartora E. G. da Silva Miozzo I. C. de Barba M. E. F.. (2011). Validation of the Brazilian Portuguese version of the Pittsburgh Sleep Quality Index. Sleep Med. 12, 70–75. doi: 10.1016/j.sleep.2010.04.020, PMID: 21145786

[ref14] Bertolazi A. N. Fagondes S. C. Hoff L. S. Pedro V. D. Barreto S. S. M. Johns M. W. (2009). Portuguese-language version of the Epworth sleepiness scale: validation for use in Brazil. J. Bras. Pneumol. 35, 877–883. doi: 10.1590/s1806-37132009000900009, PMID: 19820814

[ref15] Bicalho C. C. F. Melo G. F. Noce F. (2020). Resilience of athletes: a systematic review based on a citation network analysis. Cuad. Psicol. Deporte 20, 26–40. doi: 10.6018/cpd.391581

[ref16] Bowen L. Gross A. S. Gimpel M. Li F. X. (2017). Accumulated workloads and the acute: chronic workload ratio relate to injury risk in elite youth football players. Br. J. Sports Med. 51, 452–459. doi: 10.1136/bjsports-2015-095820, PMID: 27450360 PMC5460663

[ref17] Brink M. S. Visscher C. Arends S. Zwerver J. Post W. J. Lemmink K. A. (2010). Monitoring stress and recovery: new insights for the prevention of injuries and illnesses in elite youth soccer players. Br. J. Sports Med. 44, 809–815. doi: 10.1136/bjsm.2009.069476, PMID: 20511621

[ref18] Chang C. Putukian M. Aerni G. Diamond A. Hong G. Ingram Y.. (2020). Mental health issues and psychological factors in athletes: detection, management, effect on performance and prevention: American Medical Society for Sports Medicine Position Statement-Executive Summary. Br. J. Sports Med. 54, 216–220. doi: 10.1136/bjsports-2019-101583, PMID: 31810972

[ref19] Claudino J. G. Cardoso Filho C. A. Boullosa D. Lima-Alves A. Carrion G. R. da Silva Gianoni R. L.. (2021). The role of veracity on the load monitoring of professional soccer players: a systematic review in the face of the big data era. Appl. Sci. 11:6479. doi: 10.3390/app11146479

[ref20] Clemente F. M. Afonso J. Costa J. Oliveira R. Pino-Ortega J. Rico-González M. (2021a). Relationships between sleep, athletic and match performance, training load, and injuries: a systematic review of soccer players. Healthcare 9:808. doi: 10.3390/healthcare9070808, PMID: 34206948 PMC8305909

[ref21] Clemente F. M. Praça G. M. Aquino R. Castillo J. R.-G. Raya-González J. Rico-González M.. (2023). Effects of pitch size on soccer players’ physiological, physical, technical, and tactical responses during small-sided games: a meta-analytical comparison. Biol. Sport 40, 111–147. doi: 10.5114/biolsport.2023.110748, PMID: 36636192 PMC9806761

[ref22] Clemente F. M. Ramirez-Campillo R. Castillo D. Raya-González J. Silva A. F. Afonso J.. (2021b). Effects of mental fatigue in total running distance and tactical behavior during small-sided games: a systematic review with a meta-analysis in youth and young Adult’s soccer players. Front. Psychol. 12:656445. doi: 10.3389/fpsyg.2021.656445, PMID: 33815237 PMC8009995

[ref23] Clemente-Suárez V. J. Fuentes-García J. P. de la Vega Marcos R. Martínez Patiño M. J. (2020). Modulators of the personal and professional threat perception of Olympic athletes in the actual COVID-19 crisis. Front. Psychol. 11:1985. doi: 10.3389/fpsyg.2020.01985, PMID: 32849157 PMC7419607

[ref24] Coimbra D. R. Bevilacqua G. G. Pereira F. S. Andrade A. (2021). Effect of mindfulness training on fatigue and recovery in elite volleyball athletes: a randomized controlled follow-up study. J. Sports Sci. Med. 20, 1–8. doi: 10.52082/jssm.2021.1, PMID: 33707980 PMC7919357

[ref25] D’Oliveira A. De Souza L. C. Langiano E. Falese L. Diotaiuti P. Vilarino G. T.. (2022). Home physical exercise protocol for older adults, applied remotely during the COVID-19 pandemic: protocol for randomized and controlled trial. Front. Psychol. 13:828495. doi: 10.3389/fpsyg.2022.828495, PMID: 35185739 PMC8855123

[ref26] da Cruz W. M. D’ Oliveira A. Dominski F. H. Diotaiuti P. Andrade A. (2022). Mental health of older people in social isolation: the role of physical activity at home during the COVID-19 pandemic. Sport Sci. Health 18, 597–602. doi: 10.1007/s11332-021-00825-9, PMID: 34457072 PMC8386142

[ref27] de Rose Junior D. Vasconcellos E. G. (1997). Ansiedade-traço competitiva e atletismo: Um estudo com atletas infanto-juvenis. Rev. Paul. Educ. Fís. 11, 148–157. doi: 10.11606/issn.2594-5904.rpef.1997.138565

[ref28] Di Fronso S. Nakamura F. Y. Bortoli L. Robazza C. Bertollo M. (2013). Stress and recovery balance in amateur basketball players: differences by gender and preparation phase. Int. J. Sports Physiol. Perform. 8, 618–622. doi: 10.1123/ijspp.8.6.618, PMID: 23479432

[ref29] Diotaiuti P. Valente G. Mancone S. Corrado S. Bellizzi F. Falese L.. (2023). Effects of cognitive appraisals on perceived self-efficacy and distress during the COVID-19 lockdown: an empirical analysis based on structural equation modeling. Int. J. Environ. Res. Public Health 20:5294. doi: 10.3390/ijerph20075294, PMID: 37047910 PMC10094671

[ref30] Diotaiuti P. Valente G. Mancone S. Falese L. Bellizzi F. Anastasi D.. (2021). Perception of risk, self-efficacy and social trust during the diffusion of COVID-19 in Italy. Int. J. Environ. Res. Public Health 18:3427. doi: 10.3390/ijerph18073427, PMID: 33806194 PMC8036340

[ref31] Dithurbide L. Boudreault V. Durand-Bush N. MacLeod L. Gauthier V. (2022). The impact of the COVID-19 pandemic on Canadian national team athletes’ mental performance and mental health: the perspectives of mental performance consultants and mental health practitioners. Front. Psychol. 13:937962. doi: 10.3389/fpsyg.2022.937962, PMID: 36059762 PMC9435585

[ref32] Doherty R. Madigan S. M. Nevill A. Warrington G. Ellis J. G. (2021). The sleep and recovery practices of athletes. Nutrients 13, 1–25. doi: 10.3390/nu13041330, PMID: 33920560 PMC8072992

[ref33] Dominski F. H. Serafim T. T. Siqueira T. C. Andrade A. (2021). Psychological variables of CrossFit participants: a systematic review. Sport Sci. Health 17, 21–41. doi: 10.1007/s11332-020-00685-9, PMID: 32904532 PMC7456358

[ref34] Dönmez G. Özkan Ö. Menderes Y. Torgutalp Ş. Ş. Karaçoban L. Denerel N.. (2022). The effects of home confinement on physical activity level and mental status in professional football players during COVID-19 outbreak. Phys. Sportsmed. 50, 157–163. doi: 10.1080/00913847.2021.1888630, PMID: 33593234

[ref35] Ermidis G. Randers M. B. Krustrup P. Mohr M. (2019). Technical demands across playing positions of the Asian cup in male football. Int. J. Perform. Anal. Sport 19, 530–542. doi: 10.1080/24748668.2019.1632571

[ref36] Esteves N. S. de Brito M. A. Müller V. T. Brito C. J. Pérez D. I. V. Slimani M.. (2021). COVID-19 pandemic impacts on the mental health of professional soccer: comparison of anxiety between genders. Front. Psychol. 12:765914. doi: 10.3389/fpsyg.2021.765914 34858293 PMC8631823

[ref37] Facer-Childs E. R. Hoffman D. Tran J. N. Drummond S. P. A. Rajaratnam S. M. W. (2021). Sleep and mental health in athletes during COVID-19 lockdown. Sleep 44, 1–9. doi: 10.1093/sleep/zsaa261, PMID: 33535229 PMC7928674

[ref38] Friebe D. Fischer M. Giesche F. Füzéki E. Banzer W. (2022). Effects of the COVID-19 lockdown on physical performance parameters in professional football: a narrative literature review. Zentralbl. Arbeitsmed. Arbeitsschutz Ergon. 72, 89–97. doi: 10.1007/s40664-022-00455-z, PMID: 35095213 PMC8785923

[ref39] García-Aliaga A. Marquina M. Román I. R. Solana D. M. Madrona J. A. P. Del Campo R. L.. (2022). COVID-19 confinement effects on game actions during competition restart in professional soccer players. Int. J. Environ. Res. Public Health 19:4252. doi: 10.3390/ijerph19074252, PMID: 35409933 PMC8999149

[ref40] Gledhill A. Forsdyke D. Murray E. (2018). Psychological interventions used to reduce sports injuries: a systematic review of real-world effectiveness. Br. J. Sports Med. 52, 967–971. doi: 10.1136/bjsports-2017-097694, PMID: 29463497

[ref41] Gouttebarge V. Ahmad I. Mountjoy M. Rice S. Kerkhoffs G. (2022). Anxiety and depressive symptoms during the COVID-19 emergency period: a comparative cross-sectional study in professional football. Clin. J. Sport Med. 32, 21–27. doi: 10.1097/JSM.0000000000000886, PMID: 32941374

[ref42] Gouttebarge V. Aoki H. Kerkhoffs G. (2015). Symptoms of common mental disorders and adverse health behaviours in male professional soccer players. J. Hum. Kinet. 49, 277–286. doi: 10.1515/hukin-2015-0130, PMID: 26925182 PMC4723178

[ref43] Grazioli R. Loturco I. Baroni B. M. Oliveira G. S. Saciura V. Vanoni E.. (2020). Coronavirus disease-19 quarantine is more detrimental than traditional off-season on physical conditioning of professional soccer players. J. Strength Cond. Res. 34, 3316–3320. doi: 10.1519/JSC.0000000000003890 33136774

[ref44] Gupta L. Morgan K. Gilchrist S. (2017). Does elite sport degrade sleep quality? A systematic review. Sports Med. 47, 1317–1333. doi: 10.1007/s40279-016-0650-6, PMID: 27900583 PMC5488138

[ref45] Gwyther K. Rice S. Purcell R. Pilkington V. Santesteban-Echarri O. Bailey A.. (2022). Sleep interventions for performance, mood and sleep outcomes in athletes: a systematic review and meta-analysis. Psychol. Sport Exerc. 58:102094. doi: 10.1016/j.psychsport.2021.102094

[ref46] Haddad M. Chaouachi A. Wong D. P. Castagna C. Hambli M. Hue O.. (2013). Influence of fatigue, stress, muscle soreness and sleep on perceived exertion during submaximal effort. Physiol. Behav. 119, 185–189. doi: 10.1016/j.physbeh.2013.06.016, PMID: 23816982

[ref47] Hagiwara G. Tsunokawa T. Iwatsuki T. Shimozono H. Kawazura T. (2021). Relationships among student-athletes’ identity, mental health, and social support in Japanese student-athletes during the COVID-19 pandemic. Int. J. Environ. Res. Public Health 18:7032. doi: 10.3390/ijerph18137032, PMID: 34209463 PMC8297159

[ref48] Håkansson A. Jönsson C. Kenttä G. (2020). Psychological distress and problem gambling in elite athletes during COVID-19 restrictions—a web survey in top leagues of three sports during the pandemic. Int. J. Environ. Res. Public Health 17:6693. doi: 10.3390/ijerph17186693, PMID: 32937978 PMC7559357

[ref49] Iaia M. F. Rampinini E. Bangsbo J. (2009). High-intensity training in football. Int. J. Sports Physiol. Perform. 4, 291–306. doi: 10.1123/ijspp.4.3.291 19953818

[ref50] Impellizzeri F. M. Marcora S. M. Coutts A. J. (2019). Internal and external training load: 15 years on training load: internal and external load theoretical framework: the training process. Int. J. Sports Physiol. Perform. 14, 270–273. doi: 10.1123/ijspp.2018-0935 30614348

[ref51] Ivarsson A. Kilhage-Persson A. Martindale R. Priestley D. Huijgen B. Ardern C.. (2020). Psychological factors and future performance of football players: a systematic review with meta-analysis. J. Sci. Med. Sport 23, 415–420. doi: 10.1016/j.jsams.2019.10.021, PMID: 31753742

[ref52] Jansen P. (2021). Self-compassion and repetitive thinking in relation to depressive mood and fear of the future: an investigation during the 2020 coronavirus pandemic in semiprofessional football players. Ger. J. Exerc. Sport Res. 51, 232–236. doi: 10.1007/s12662-021-00712-y PMC7930895 40477278

[ref53] Jaspers A. Brink M. S. Probst S. G. M. Frencken W. G. P. Helsen W. F. (2017). Relationships between training load indicators and training outcomes in professional soccer. Sports Med. 47, 533–544. doi: 10.1007/s40279-016-0591-0, PMID: 27459866

[ref54] Jemal M. Trabelsi K. Boukhris O. Ammar A. Clark C. C. T. Chtourou H. (2022). Sleep and daytime sleepiness in elite athletes and sedentary individuals. Sci. Sports 37, 31–36. doi: 10.1016/j.scispo.2021.02.009

[ref55] Jia L. Carter M. V. Cusano A. Li X. Kelly J. D. Bartley J. D.. (2022). The effect of the COVID-19 pandemic on the mental and emotional health of athletes: a systematic review. Am. J. Sports Med. 51, 2207–2215. doi: 10.1177/03635465221087473, PMID: 35413208 PMC10333562

[ref56] Jordana A. Turner M. J. Ramis Y. Torregrossa M. (2020). A systematic mapping review on the use of rational emotive behavior therapy (REBT) with athletes. Int. Rev. Sport Exerc. Psychol. 16, 231–256. doi: 10.1080/1750984X.2020.1836673

[ref57] Jurecka A. Skucińska P. Gądek A. (2021). Impact of the SARS-CoV-2 coronavirus pandemic on physical activity, mental health and quality of life in professional athletes—a systematic review. Int. J. Environ. Res. Public Health 18:9423. doi: 10.3390/ijerph18179423, PMID: 34502010 PMC8431129

[ref58] Katanic B. Bjelica D. Corluka M. Preljevic A. Osmani A. (2022). Motivational structure for sports practice during COVID-19 pandemic in professional football players. Sport Mont J. 20, 127–131. doi: 10.26773/smj.221020

[ref59] Keemss J. Sieland J. Pfab F. Banzer W. (2022). Effects of COVID-19 lockdown on physical performance, sleep quality, and health-related quality of life in professional youth soccer players. Front. Sports Act. Living 4:875767. doi: 10.3389/fspor.2022.875767, PMID: 35769222 PMC9234262

[ref60] Kocevska D. Blanken T. F. Van Someren E. J. W. Rösler L. (2020). Sleep quality during the COVID-19 pandemic: not one size fits all. Sleep Med. 76, 86–88. doi: 10.1016/j.sleep.2020.09.029 33126035 PMC7543757

[ref61] Kunrath C. A. da Silva Leite Cardoso F. Calvo T. G. da Costa I. T. (2020). Mental fatigue in soccer: a systematic review. Rev. Bras. Med. Esporte 26, 172–178. doi: 10.1590/1517-869220202602208206

[ref62] Kurniarobbi J. Chikih C. Andeansah M. Lestari R. Sukendar I. (2022). Athletes sleep duration during COVID-19 pandemic and its relationship with health condition. Int. J. Public Health Sci. 11, 61–68. doi: 10.11591/ijphs.v11i1.21089

[ref63] Lastella M. Roach G. D. Halson S. L. Sargent C. (2016). The chronotype of elite athletes. J. Hum. Kinet. 54, 219–225. doi: 10.1515/hukin-2016-0049, PMID: 28031772 PMC5187972

[ref64] Laux P. Krumm B. Diers M. Flor H. (2015). Recovery-stress balance and injury risk in professional football players: a prospective study. J. Sports Sci. 33, 2140–2148. doi: 10.1080/02640414.2015.1064538, PMID: 26168148 PMC4673559

[ref65] Leguizamo F. Olmedilla A. Núñez A. Verdaguer F. J. P. (2021). Personality, coping strategies, and mental health in high-performance athletes during confinement derived from the COVID-19 pandemic. Front. Public Health 8:561198. doi: 10.3389/fpubh.2020.561198, PMID: 33490009 PMC7820785

[ref66] Lima Y. Denerel N. Öz N. D. Senisik S. (2021). The psychological impact of COVID-19 infection on athletes: example of professional male football players. Sci. Med. Footb. 5, 53–61. doi: 10.1080/24733938.2021.1933156, PMID: 35077314

[ref67] Lin C. H. Lu F. J. H. Chen T. W. Hsu Y. (2022). Relationship between athlete stress and burnout: a systematic review and meta-analysis. Int. J. Sport Exerc. Psychol. 20, 1295–1315. doi: 10.1080/1612197X.2021.1987503

[ref68] Lin Y. Mutz J. Clough P. J. Papageorgiou K. A. (2017). Mental toughness and individual differences in learning, educational and work performance, psychological well-being, and personality: a systematic review. Front. Psychol. 8:1345. doi: 10.3389/fpsyg.2017.01345, PMID: 28848466 PMC5554528

[ref69] Listiyandini R. A. Andriani A. Afsari N. Krisnamurthi P. B. U. Moulds M. L. Mahoney A. E. J.. (2024). Evaluating the feasibility of a guided culturally adapted internet-delivered mindfulness intervention for Indonesian university students experiencing psychological distress. Mindfulness 15, 1095–1108. doi: 10.1007/s12671-024-02346-1

[ref70] Liu Z. Zhao L. Wang S. Gao Y. Zhang L. (2022). The association between occupational stress and mental health among Chinese soccer referees in the early stage of reopening soccer matches during the COVID-19 pandemic outbreak: a moderated mediation model. Int. J. Environ. Res. Public Health 19:16750. doi: 10.3390/ijerph192416750, PMID: 36554631 PMC9778837

[ref71] Madsen E. E. Krustrup P. Larsen C. H. Elbe A. M. Wikman J. M. Ivarsson A.. (2021). Resilience as a protective factor for well-being and emotional stability in elite-level football players during the first wave of the COVID-19 pandemic. Sci. Med. Footb. 5, 62–69. doi: 10.1080/24733938.2021.1959047, PMID: 35077313

[ref72] Martínez-Patiño M. J. Lopez F. J. B. Dubois M. Vilain E. Fuentes-García J. P. (2021). Effects of COVID-19 home confinement on behavior, perception of threat, stress and training patterns of Olympic and Paralympic athletes. Int. J. Environ. Res. Public Health 18:12780. doi: 10.3390/ijerph182312780, PMID: 34886503 PMC8656930

[ref73] Meeusen R. Nederhof E. Buyse L. Roelands B. De Schutter G. Piacentini M. F. (2010). Diagnosing overtraining in athletes using the twobout exercise protocol. Br. J. Sports Med. 44, 642–648. doi: 10.1136/bjsm.2008.049981, PMID: 18703548

[ref74] Mehrsafar A. H. Moghadam Zadeh A. Gazerani P. Jaenes Sanchez J. C. Nejat M. Rajabian Tabesh M.. (2021). Mental health status, life satisfaction, and mood state of elite athletes during the COVID-19 pandemic: a follow-up study in the phases of home confinement, reopening, and semi-lockdown condition. Front. Psychol. 12:630414. doi: 10.3389/fpsyg.2021.630414, PMID: 34177691 PMC8231927

[ref75] Melone M. A. Tourny C. Gehlbach B. K. Schmidt E. L. Lalevée M. L’Hermette M. (2022). Prevalence and risk factors of poor sleep quality in collegiate athletes during COVID-19 pandemic: a cross-sectional study. Int. J. Environ. Res. Public Health 19:3098. doi: 10.3390/ijerph19053098, PMID: 35270790 PMC8910097

[ref76] Merino-Muñoz P. Pérez-Contreras J. Adasme-Maureira F. Aedo-Muñoz E. (2021). Efectos en el estado de bienestar en periodo de confinamiento debido al COVID-19 en jugadores profesionales de fútbol. MHSalud 19, 1–12. doi: 10.15359/mhs.19-1.2

[ref77] Miguel M. Oliveira R. Loureiro N. García-Rubio J. Ibáñez S. J. (2021). Load measures in training/match monitoring in soccer: a systematic review. Int. J. Environ. Res. Public Health 18:2721. doi: 10.3390/ijerph18052721, PMID: 33800275 PMC7967450

[ref78] Mohr M. Nassis G. P. Brito J. Randers M. B. Castagna C. Parnell D.. (2020). Return to elite football after the COVID-19 lockdown. Manag. Sport Leis. 27, 172–180. doi: 10.1080/23750472.2020.1768635

[ref79] Mon-López D. García-Aliaga A. Ginés Bartolomé A. Muriarte Solana D. (2020). How has COVID-19 modified training and mood in professional and non-professional football players? Physiol. Behav. 227:113148. doi: 10.1016/j.physbeh.2020.113148, PMID: 32858031 PMC7445487

[ref80] Mota G. R. Santos I. A. Marocolo M. (2021). Change in soccer substitutions rule due to COVID-19: why only five substitutions? Front. Sports Act. Living 2:588369. doi: 10.3389/fspor.2020.588369, PMID: 33521632 PMC7844060

[ref81] Orviz-Martínez N. Botey-Fullat M. Arce-García S. (2021). Analysis of burnout and psychosocial factors in grassroot football referees. Int. J. Environ. Res. Public Health 18:1111. doi: 10.3390/ijerph18031111, PMID: 33513797 PMC7908562

[ref82] Parsak B. Saraç L. (2022). Social distancing and quality of life among candidates for the sports science degree during the COVID-19 pandemic. Pamukkale J. Sport Sci. 13, 52–69. doi: 10.54141/psbd.1084588

[ref83] Pellino V. C. Lovecchio N. Puci M. V. Marin L. Gatti A. Pirazzi A.. (2022). Effects of the lockdown period on the mental health of elite athletes during the COVID-19 pandemic: a narrative review. Sport Sci. Health 18, 1187–1199. doi: 10.1007/s11332-022-00964-7, PMID: 35693326 PMC9174028

[ref84] Pété E. Leprince C. Lienhart N. Doron J. (2022). Dealing with the impact of the COVID-19 outbreak: are some athletes’ coping profiles more adaptive than others? Eur. J. Sport Sci. 22, 237–247. doi: 10.1080/17461391.2021.1873422, PMID: 33410729

[ref85] Pillay L. van Rensburg DCC J. Jansen van Rensburg A. Ramagole D. A. Holtzhausen L. Dijkstra H. P.. (2020). Nowhere to hide: the significant impact of coronavirus disease 2019 (COVID-19) measures on elite and semi-elite South African athletes. J. Sci. Med. Sport 23, 670–679. doi: 10.1016/j.jsams.2020.05.016, PMID: 32448749 PMC7235602

[ref86] Poitras V. J. Gray C. E. Borghese M. Carson V. Chaput J.-P. Jansen I.. (2016). Systematic review of the relationships between objectively measured physical activity and health indicators in school-aged children and youth. Appl. Physiol. Nutr. Metab. 41, S266–S282. doi: 10.1139/apnm-2015-0627, PMID: 27306431

[ref87] Polito L. F. T. Figueira A. J. Miranda M. L. J. Chtourou H. Miranda J. M. Brandão M. R. F. (2017). Psychophysiological indicators of fatigue in soccer players: a systematic review. Sci. Sports 32, 1–13. doi: 10.1016/j.scispo.2016.09.003

[ref88] Pons J. Ramis Y. Alcaraz S. Jordana A. Borrueco M. Torregrossa M. (2020). Where did all the sport go? negative impact of COVID-19 lockdown on life-spheres and mental health of Spanish young athletes. Front. Psychol. 11:611872. doi: 10.3389/fpsyg.2020.611872, PMID: 33365006 PMC7750436

[ref89] Reilly T. Edwards B. (2007). Altered sleep-wake cycles and physical performance in athletes. Physiol. Behav. 90, 274–284. doi: 10.1016/j.physbeh.2006.09.017, PMID: 17067642

[ref90] Robey E. Dawson B. Halson S. Gregson W. Goodman C. Eastwood P. (2014). Sleep quantity and quality in elite youth soccer players: a pilot study. Eur. J. Sport Sci. 14, 410–417. doi: 10.1080/17461391.2013.843024, PMID: 24093813

[ref91] Romdhani M. Fullagar H. H. K. Vitale J. A. Nédélec M. Rae D. E. Ammar A.. (2022a). Lockdown duration and training intensity affect sleep behavior in an international sample of 1,454 elite athletes. Front. Physiol. 13:904778. doi: 10.3389/fphys.2022.904778, PMID: 35784859 PMC9240664

[ref92] Romdhani M. Rae D. E. Nédélec M. Ammar A. Chtourou H. Al Horani R.. (2022b). COVID-19 lockdowns: a worldwide survey of circadian rhythms and sleep quality in 3,911 athletes from 49 countries, with data-driven recommendations. Sports Med. 52, 1433–1448. doi: 10.1007/s40279-021-01601-y, PMID: 34878639 PMC8652380

[ref93] Romdhani M. Washif J. A. Taylor L. Chamari K. Ammar A. Al Horani R.. (2023). Soccer players’ sleep quality and training load were affected by the COVID-19 lockdown: an international survey. Int. J. Sports Physiol. Perform. 18, 530–540. doi: 10.1123/IJSPP.2022-0187, PMID: 37030665

[ref94] Rupprecht A. G. O. Tran U. S. Gröpel P. (2021). The effectiveness of pre-performance routines in sports: a meta-analysis. Int. Rev. Sport Exerc. Psychol. 17, 39–64. doi: 10.1080/1750984X.2021.1944271

[ref95] Russell S. Jenkins D. Rynne S. Halson S. L. Kelly V. (2019). What is mental fatigue in elite sport? Perceptions from athletes and staff. Eur. J. Sport Sci. 19, 1367–1376. doi: 10.1080/17461391.2019.1618397, PMID: 31081474

[ref96] Sargent C. Lastella M. Halson S. L. Roach G. D. (2021). How much sleep does an elite athlete need? Int. J. Sports Physiol. Perform. 16, 1746–1757. doi: 10.1123/ijspp.2020-0896, PMID: 34021090

[ref97] Sarmento H. Frontini R. Marques A. Peralta M. Ordoñez-Saavedra N. Duarte J. P.. (2021). Depressive symptoms and burnout in football players: a systematic review. Brain Sci. 11:1351. doi: 10.3390/brainsci11101351, PMID: 34679415 PMC8534279

[ref98] Saw A. E. Main L. C. Gastin P. B. (2016). Monitoring the athlete training response: subjective self-reported measures trump commonly used objective measures: a systematic review. Br. J. Sports Med. 50, 281–291. doi: 10.1136/bjsports-2015-094758, PMID: 26423706 PMC4789708

[ref99] Sebri V. Pizzoli S. F. M. Pravettoni G. (2024). What does my anxiety look like? A thematic analysis of the impact of a single session imagery technique on emotional issues. J. Ration. - Emot. Cogn. - Behav. Ther. 42, 780–795. doi: 10.1007/s10942-024-00545-2

[ref100] Slimani M. Baker J. S. Cheour F. Taylor L. Bragazzi N. L. (2017). Steroid hormones and psychological responses to soccer matches: insights from a systematic review and meta-analysis. PLoS One 12:e0186100. doi: 10.1371/journal.pone.0186100, PMID: 29023546 PMC5638322

[ref101] Tan C. Wang J. Yin J. Cao G. Cao L. Chen C.. (2023). The effect of prolonged closed-loop management on athletes’ sleep and mood during COVID-19 pandemic: evidence from the 2022 Shanghai Omicron Wave. PLoS One 18:e0284858. doi: 10.1371/journal.pone.0284858, PMID: 37079590 PMC10118198

[ref102] Thapa R. K. Lum D. Moran J. Ramirez-Campillo R. (2021). Effects of complex training on Sprint, jump, and change of direction ability of soccer players: a systematic review and meta-analysis. Front. Psychol. 11:627869. doi: 10.3389/fpsyg.2020.627869, PMID: 33551937 PMC7862112

[ref103] Thompson C. J. Noon M. Towlson C. Perry J. Coutts A. J. Harper L. D.. (2020). Understanding the presence of mental fatigue in English academy soccer players. J. Sports Sci. 38, 1524–1530. doi: 10.1080/02640414.2020.1746597 32212903

[ref104] Trabelsi K. Ammar A. Glenn J. M. Boukhris O. Khacharem A. Bouaziz B.. (2022). Does observance of Ramadan affect sleep in athletes and physically active individuals? A systematic review and meta-analysis. J. Sleep Res. 31, e13503–e13521. doi: 10.1111/jsr.13503, PMID: 34693577

[ref105] Villaseca-Vicuña R. Pérez-Contreras J. Merino-Muñoz P. González-Jurado J. A. Aedo-Muñoz E. (2021). Effects of COVID-19 confinement measures on training loads and the level of well-being in players from Chile women’s national soccer team. Rev. Fac. Med. 69, 1–7. doi: 10.15446/revfacmed.v69n1.88480

[ref106] Vindegaard N. Benros M. E. (2020). COVID-19 pandemic and mental health consequences: systematic review of the current evidence. Brain Behav. Immun. 89, 531–542. doi: 10.1016/j.bbi.2020.05.048, PMID: 32485289 PMC7260522

[ref107] Wagemans J. Catteeuw P. Vandenhouten J. Jansen J. de Corte X. Ceusters C.. (2021). The impact of COVID-19 on physical performance and mental health—a retrospective case series of Belgian male professional football players. Front. Sports Act. Living 3:803130. doi: 10.3389/fspor.2021.803130 34966896 PMC8710515

[ref108] Walsh N. P. Halson S. L. Sargent C. Roach G. D. Nédélec M. Gupta L.. (2021). Sleep and the athlete: narrative review and 2021 expert consensus recommendations. Br. J. Sports Med. 55, 356–368. doi: 10.1136/bjsports-2020-102025, PMID: 33144349

[ref109] Walsh A. Harris S. Beranek P. Vial S. Cruickshank T. Turner M. (2022). Effect of physical activity during COVID-19 on the sleep health of community-level athletes in Australia. Sport Sci. Health 18, 1475–1481. doi: 10.1007/s11332-022-00947-8, PMID: 35669926 PMC9154202

[ref110] Zhang S. Cao J. Ahn C. (2014). A GEE approach to determine sample size for pre- and post-intervention experiments with dropout. Comput. Stat. Data Anal. 69, 114–121. doi: 10.1016/j.csda.2013.07.037, PMID: 24293779 PMC3842849

[ref111] Zinner C. Matzka M. Leppich R. Kounev S. Holmberg H.-C. Sperlich B. (2020). The impact of the German strategy for containment of coronavirus SARS-CoV-2 on training characteristics, physical activity and sleep of highly trained kayakers and canoeists: a retrospective observational study. Front. Sports Act. Living 2:579830. doi: 10.3389/fspor.2020.579830, PMID: 33345147 PMC7739795

